# Exposure to Moderate Air Pollution during Late Pregnancy and Cord Blood Cytokine Secretion in Healthy Neonates

**DOI:** 10.1371/journal.pone.0023130

**Published:** 2011-08-03

**Authors:** Philipp Latzin, Urs Frey, Jakob Armann, Elisabeth Kieninger, Oliver Fuchs, Martin Röösli, Bianca Schaub

**Affiliations:** 1 Division of Respiratory Medicine, Department of Paediatrics, Inselspital, University of Bern, Bern, Switzerland; 2 University Children's Hospital (UKBB), University of Basel, Basel, Switzerland; 3 Department of Allergy & Pulmonary, University Children's Hospital Munich, Ludwig Maximilian University of Munich (LMU), Munich, Germany; 4 Swiss Tropical and Public Health Institute, University of Basel, Basel, Switzerland; 5 University of Basel, Basel, Switzerland; Ludwig-Maximilians-Universität München, Germany

## Abstract

**Background/Objectives:**

Ambient air pollution can alter cytokine concentrations as shown *in vitro* and following short-term exposure to high air pollution levels *in vivo*. Exposure to pollution during late pregnancy has been shown to affect fetal lymphocytic immunophenotypes. However, effects of prenatal exposure to moderate levels of air pollutants on cytokine regulation in cord blood of healthy infants are unknown.

**Methods:**

In a birth cohort of 265 healthy term-born neonates, we assessed maternal exposure to particles with an aerodynamic diameter of 10 µm or less (PM_10_), as well as to indoor air pollution during the last trimester, specifically the last 21, 14, 7, 3 and 1 days of pregnancy. As a proxy for traffic-related air pollution, we determined the distance of mothers' homes to major roads. We measured cytokine and chemokine levels (MCP-1, IL-6, IL-10, IL-1ß, TNF-α and GM-CSF) in cord blood serum using LUMINEX technology. Their association with pollution levels was assessed using regression analysis, adjusted for possible confounders.

**Results:**

Mean (95%-CI) PM_10_ exposure for the last 7 days of pregnancy was 18.3 (10.3–38.4 µg/m^3^). PM_10_ exposure during the last 3 days of pregnancy was significantly associated with reduced IL-10 and during the last 3 months of pregnancy with increased IL-1ß levels in cord blood after adjustment for relevant confounders. Maternal smoking was associated with reduced IL-6 levels. For the other cytokines no association was found.

**Conclusions:**

Our results suggest that even naturally occurring prenatal exposure to moderate amounts of indoor and outdoor air pollution may lead to changes in cord blood cytokine levels in a population based cohort.

## Introduction

A wide range of epidemiological studies indicates that exposure to air pollution is associated with respiratory morbidities including allergic diseases [1], [2], [3]. So far, in these studies ambient air particles, especially particles with an aerodynamic diameter of 10 µm or less (PM10), as well as ozone (O_3_) and nitrogen dioxide (NO_2_) have been found responsible for the observed health effects. While associations of respiratory morbidity and activation of the immune system after short-term exposure to high levels of pollution in individuals are well examined [4], effects of long-term exposure to moderate levels of air pollution on a population level have not been well studied, and the underlying biological mechanisms are still unclear. Potential immune mechanisms involved include a change in alveolar macrophage function *in vitro* or an induction of pro- and anti-inflammatory cytokines and chemokines, as confirmed *in vivo* in adults exposed to high amounts of air pollutants [5], [6].

Compared to studies in subjects with a mature immune system, influences on neonatal immune maturation develop over a longer time period and thus exposure to moderate levels of air pollution play a role. In fact there is increasing evidence suggesting that the development of immunological responses in childhood may be influenced by early - and most likely in utero - responses to a selection of stimuli [7], [8], [9]. In this context, early life exposures to environmental stimuli have been suggested to modulate a child's immune system which may influence the subsequent development of allergic diseases [7]. While distinct environmental exposure in utero such as microbial stimuli may be protective for the development of allergic diseases in childhood via specific immune regulatory mechanisms [8], [10], exposure to air pollution was shown to be associated with an increase in allergic diseases [11]. Indeed, first evidence indicates influences on cord blood immune responses such as a change in relative distribution of lymphocytes by exposure to low ambient air pollution or if mothers smoked during pregnancy [12], [13].

In this study, we aimed to assess the influence of maternal exposure to moderate outdoor and indoor air pollution over different time periods during the last trimester of pregnancy on early immune responses in cord blood. Specifically, we assessed expression of the pro-inflammatory cytokines interleukin (IL-)6, IL-1ß, granulocyte macrophage colony-stimulating factor (GM-CSF), Tumor necrosis factor α (TNF-α) and the anti-inflammatory cytokine IL-10, in addition to expression of the pro-inflammatory chemokine monocyte chemotactic protein-1 (MCP-1/CCL2) in cord blood serum in a cohort of healthy term-born neonates. Those markers have been shown to be elevated in subjects exposed to high levels of PM_10_ after an episode of acute air pollution, indicating the induction of a systematic inflammatory response [6], [14].

## Results

The study enrolled 265 infants, with complete data from 199 (75%) available. From 66 infants no cord blood samples were collected, mainly due to logistical reasons (birth on the weekend, no cord blood available or stored by the parents for other purposes). Anthropometric data and air pollution exposure are given in Table 1, cord blood cytokine concentrations and distribution of possible risk factors are given in Table 2.

**Table 1:** Demographics and exposure to air pollution of the study infants.

	Median	Interquartile Range (IQR)	Range
Anthropometric data (n = 199)			
Gestational age at birth, wks	40.0	39.1–40.9	37.0–42.3
Birth weight, kg	3.4	3.1–3.7	2.5–4.9
Exposure to air pollution			
Mean PM_10_, third trimester, µg/m^3^	20.1	17.2–24.3	14.4–37.2
Mean PM_10_, last 21 days, µg/m^3^	19.8	16.4–24.5	11.0–74.6
Mean PM_10_, last 14 days, µg/m^3^	19.8	15.6–24.2	8.8–94.4
Mean PM_10_, last 7 days, µg/m^3^	18.2	14.9–23.6	7.4–82.9
Mean PM_10_, last 3 days, µg/m^3^	17.8	12.6–25.1	5.2–71.0
Mean PM_10_, last 1 day, µg/m^3^	18.5	12.0–26.5	3.0–90.1
Distance to major roads (6 m wide), m^1^	166	65–368	3–3794
Maternal smoking during pregnancy, no. (%)		21/199 (11)	
Smoking of the father, no. (%)		48/199 (24)	
Gas cooking at home, no. (%)^2^		15/174 (9)	
Open fireplace at home, no. (%)^2^		57/174 (33)	
Any indoor pollution, no. (%)^3^		107/199 (54)	

1 for one subject, this could not be determined, as no GIS data were available.

2 information on gas cooking and open fireplace was available only from 174 subjects.

3 defined as exposure to either maternal smoking during pregnancy, smoking of the father, gas cooking or open fireplace at home.

**Table 2:** Cord blood cytokines & chemokines and possible confounders of study infants.

Cord blood cytokine and chemokine concentrations and detection rates 1			
	Median	Interquartile Range (IQR)	Detection rate
MCP-1, pg/ml	4.08	1.02–14.2	79%
IL-6, pg/ml	0.17	0.03–0.77	29%
IL-10, pg/ml	0.32	0.17–0.60	25%
IL-1ß, pg/ml	0.05	0.01–0.24	13%
TNF-α, pg/ml	0.10	0.03–0.30	5%
GM-CSF, pg/ml	0.001	<0.001–0.004	2%

Potential confounders	Number (%)
Male sex, no. (%)	112 (56)
Maternal atopy, no. (%)	79 (40)
Low maternal education, no. (%)^2^	75 (39)
High maternal education, no. (%)^2^	61 (31)
Low paternal education, no. (%)^2^	49 (25)
High paternal education, no. (%)^2^	90 (47)

1 cytokine and chemokine concentrations were determined using regression on order statistics.

2 parental education was categorized into low (less than four year of apprenticeship), middle (at least four years of apprenticeship) and high (tertiary education).

### Cord blood cytokine and chemokine concentrations

Cytokine concentrations in cord blood serum are shown in Table 2. The respective cytokines were detectable at different proportions using the two cut-offs as described. With the detection limit of the assay as cut-off, MCP-1 was well detectable in 79%, IL-6 in 29%, IL-10 in 25% and IL-1β in 13% of samples. The other markers were expressed at lower concentrations; TNF-α secretion was detectable in 5% and GM-CSF in 2% of samples. We thus decided to confine the further analysis to MCP-1, IL-6, IL-10 and IL-1β.

### Outdoor air pollution

**Figure 1:**
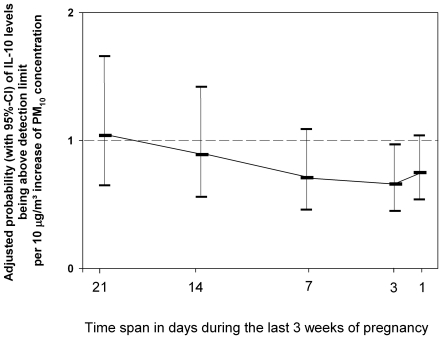
Association between outdoor pollution level during different time spans of the last 2 weeks of pregnancy and IL-10 concentrations in cord blood. Data is given as the adjusted probability (with 95%-CI) of IL-10 levels being above detection limit per 10 µg/m^3^ increase of PM_10_ concentration during the respective time spans.

Results are given in detail in Table 3, 4, 5 and 6 for the associations between pollution exposures and levels of MCP-1, IL-6, IL-10 and IL-1β, respectively. Overall, no strong association was found between exposure to air pollution during most times of pregnancy or living at major roads and the cytokine concentrations. However, for exposure to PM_10_ during the last week of pregnancy we found a trend towards lower IL-6 and IL-10 levels, which was strongest for the adjusted associations between air pollution during the last three days of pregnancy and IL-10 levels (OR 0.66, 95%-CI 0.45–0.97, p-value of 0.035, Table 5 and Figure 1). In addition, we found an association between PM_10_ during the last three months of pregnancy and elevated IL-1β levels in cord blood, with the strongest association in the adjusted model (OR 3.00, 95%-CI 1.30–6.91, p-value of 0.010, Table 6). No effect of living closer than 50 m to major roads as a proxy for traffic-related air pollution on cord blood cytokine levels was found (Tables 3 to [Table pone-0023130-t004] [Table pone-0023130-t005] 6).

**Table 3:** Associations between prenatal exposure to air pollution and cord blood concentration of MCP-1.

	Univariable model			Adjusted model^1^			Adjusted model^2^		
	OR	CI 95%	p-value	OR	CI 95%	p-value	OR	CI 95%	p-value
PM_10_ exposure, per 10 µg/m^3^									
PM_10_, last trimester	0.98	0.49–1.94	0.944	1.09	0.52–2.27	0.824	1.08	0.50–2.31	0.851
PM_10_, last 21 days	1.01	0.65–1.56	0.970	0.94	0.60–1.48	0.796	0.80	0.49–1.32	0.386
PM_10_, last 14 days	1.01	0.69–1.49	0.947	0.97	0.65–1.44	0.878	0.81	0.51–1.27	0.357
PM_10_, last 7 days	1.16	0.78–1.73	0.453	1.14	0.76–1.72	0.521	1.05	0.69–1.61	0.814
PM_10_, last 3 days	1.12	0.79–1.58	0.526	1.09	0.76–1.55	0.644	1.02	0.72–1.45	0.919
PM_10_, last 1 days	1.13	0.82–1.55	0.452	1.10	0.80–1.51	0.573	1.04	0.76–1.43	0.811
Living at a major road	1.44	0.56–3.74	0.453	1.42	0.54–3.72	0.471	1.32	0.50–3.48	0.581
Indoor air pollution									
Mother smoking	0.81	0.28–2.36	0.701	0.81	0.28–2.38	0.706	0.77	0.22–2.68	0.681
Father smoking	1.03	0.46–2.29	0.941	1.09	0.45–2.62	0.856	0.91	0.37–2.26	0.843
Gas cooking	0.97	0.26–3.64	0.963	0.93	0.24–3.52	0.913	1.07	0.27–4.21	0.927
Open fireplace	1.02	0.46–2.28	0.955	0.99	0.43–2.27	0.977	0.86	0.37–2.04	0.740

Data is given as the probability of MCP-1 being above detection limit per 10 µg/m^3^ increase of the higher PM_10_ concentration, for living within 50 m of a major road and for being exposed to indoor air pollutant.

1 this model was adjusted for gender, gestational weight and maternal smoking during pregnancy.

2 this model was further adjusted for parental education, maternal atopy and gestational age.

**Table 4 pone-0023130-t004:** Associations between prenatal exposure to air pollution and cord blood concentration of IL-6.

	Univariable model			Adjusted model^1^			Adjusted model^2^		
	OR	CI 95%	p-value	OR	CI 95%	p-value	OR	CI 95%	p-value
PM_10_ exposure, per 10 µg/m^3^									
PM_10_, last trimester	0.83	0.44–1.56	0.571	0.81	0.42–1.55	0.522	0.88	0.45–1.73	0.706
PM_10_, last 21 days	1.02	0.70–1.50	0.916	0.97	0.65–1.45	0.868	1.08	0.67–1.72	0.754
PM_10_, last 14 days	0.91	0.63–1.31	0.599	0.86	0.58–1.28	0.457	0.95	0.60–1.51	0.824
PM_10_, last 7 days	0.72	0.49–1.07	0.108	0.71	0.48–1.06	0.091	0.75	0.49–1.14	0.179
PM_10_, last 3 days	0.83	0.60–1.14	0.239	0.83	0.60–1.14	0.240	0.84	0.60–1.19	0.326
PM_10_, last 1 days	0.89	0.67–1.17	0.387	0.88	0.67–1.17	0.390	0.88	0.65–1.19	0.414
Living at a major road	1.08	0.49–2.36	0.853	1.15	0.52–2.57	0.733	1.04	0.45–2.40	0.920
Indoor air pollution									
Mother smoking	0.24	0.05–1.05	0.057	0.24	0.05–1.05	0.058	0.18	0.04–0.86	0.032
Father smoking	1.06	0.52–2.17	0.876	1.47	0.68–3.19	0.333	1.27	0.57–2.87	0.560
Gas cooking	1.31	0.42–4.04	0.642	1.17	0.38–3.64	0.784	1.13	0.34–3.73	0.841
Open fireplace	1.45	0.73–2.89	0.291	1.26	0.62–2.56	0.529	1.05	0.49–2.22	0.909

Data is given as the probability of IL-6 being above detection limit per 10 µg/m^3^ increase of the higher PM_10_ concentration, for living within 50 m of a major road and for being exposed to indoor air pollutant.

1 this model was adjusted for gender, gestational weight and maternal smoking during pregnancy.

2 this model was further adjusted for parental education, maternal atopy and gestational age.

**Table 5 pone-0023130-t005:** Associations between prenatal exposure to air pollution and cord blood concentration of IL-10.

	Univariable model			Adjusted model^1^			Adjusted model^2^		
	OR	CI 95%	p-value	OR	CI 95%	p-value	OR	CI 95%	p-value
PM_10_ exposure, per 10 µg/m^3^									
PM_10_, last trimester	0.87	0.46–1.64	0.668	0.80	0.42–1.54	0.507	0.79	0.40–1.57	0.499
PM_10_, last 21 days	1.02	0.69–1.51	0.924	0.93	0.62–1.40	0.725	1.04	0.65–1.66	0.873
PM_10_, last 14 days	0.92	0.63–1.33	0.658	0.84	0.56–1.26	0.401	0.89	0.56–1.42	0.625
PM_10_, last 7 days	0.75	0.51–1.11	0.156	0.73	0.49–1.07	0.110	0.71	0.46–1.09	0.114
PM_10_, last 3 days	0.72	0.51–1.03	0.074	0.70	0.49–1.00	0.049	0.66	0.45–0.97	0.035
PM_10_, last 1 days	0.83	0.62–1.11	0.215	0.80	0.59–1.08	0.146	0.75	0.54–1.04	0.082
Living at a major road	1.00	0.43–2.30	0.996	1.06	0.45–2.48	0.891	1.02	0.43–2.43	0.968
Indoor air pollution									
Mother smoking	0.29	0.07–1.31	0.108	0.31	0.07–1.37	0.121	0.30	0.06–1.44	0.134
Father smoking	0.78	0.35–1.71	0.527	0.96	0.41–2.21	0.916	0.87	0.37–2.08	0.763
Gas cooking	0.19	0.02–1.46	0.110	0.17	0.02–1.36	0.095	0.16	0.02–1.30	0.087
Open fireplace	2.26	1.12–4.56	0.023	1.96	0.95–4.03	0.068	1.80	0.84–3.83	0.128

Data is given as the probability of IL-10 being above detection limit per 10 µg/m^3^ increase of the higher PM_10_ concentration, for living within 50 m of a major road and for being exposed to indoor air pollutant.

1 this model was adjusted for gender, gestational weight and maternal smoking during pregnancy.

2 this model was further adjusted for parental education, maternal atopy and gestational age.

**Table 6:** Associations between prenatal exposure to air pollution and cord blood concentration of IL-1β.

	Univariable model			Adjusted model^1^			Adjusted model^2^		
	OR	CI 95%	p-value	OR	CI 95%	p-value	OR	CI 95%	p-value
PM_10_ exposure, per 10 µg/m ^3^									
PM_10_, last trimester	2.67	1.28–5.55	0.009	2.61	1.20–5.65	0.015	3.00	1.30–6.91	0.010
PM_10_, last 21 days	1.44	0.95–2.18	0.085	1.32	0.86–2.02	0.201	1.79	1.04–3.06	0.034
PM_10_, last 14 days	1.13	0.78–1.65	0.523	1.05	0.71–1.55	0.817	1.23	0.76–1.99	0.404
PM_10_, last 7 days	1.11	0.77–1.61	0.569	1.07	0.74–1.53	0.732	1.15	0.77–1.73	0.488
PM_10_, last 3 days	1.18	0.84–1.65	0.347	1.13	0.80–1.58	0.492	1.14	0.79–1.65	0.478
PM_10_, last 1 days	1.17	0.87–1.58	0.296	1.15	0.84–1.56	0.392	1.12	0.80–1.57	0.502
Living at a major road	0.35	0.08–1.55	0.166	0.37	0.08–1.65	0.190	0.39	0.08–1.82	0.231
Indoor air pollution									
Mother smoking	NA^3^								
Father smoking	0.59	0.19–1.82	0.360	0.81	0.25–2.57	0.719	0.65	0.20–2.28	0.526
Gas cooking	NA^3^								
Open fireplace	2.33	0.97–5.59	0.057	1.91	0.78–4.66	0.157	1.89	0.72–4.92	0.195

Data is given as the probability of IL-1β being above detection limit per 10 µg/m^3^ increase of the higher PM_10_ concentration, for living within 50 m of a major road and for being exposed to indoor air pollutant.

1 this model was adjusted for gender, gestational weight and maternal smoking during pregnancy.

2 this model was further adjusted for parental education, maternal atopy and gestational age.

3 these analyses could not be performed since no subject with detectable IL-1β levels was exposed.

### Indoor air pollution

No clear association was found for exposure to indoor pollution in general and most of the measured cytokines. However, when the assessed single indoor pollutants were examined separately, we found that maternal smoking during pregnancy was associated with lower IL-6 and a trend for lower IL-10 cord blood concentrations (Tables 4 and 5). The strongest association was found in the adjusted models for IL-6 (OR 0.18, 95%-CI 0.04–0.86, p-value of 0.032, Table 4). Results for other single indoor pollutants, such as gas cooking or having an open fire place were not associated with changes in cord blood cytokines, and the trend observed for open fire places at home and higher IL-10 levels disappeared after adjustment for confounders (Table 5).

All findings were confirmed by regression analysis allowing for censored data (Tobit regression and maximum likelihood estimation).

## Discussion

In this study we have investigated the association between exposure to air pollution in the last trimester of pregnancy and concentrations of pro- and anti-inflammatory cytokines and chemokines in the cord blood of newborns in a modest pollution area. After higher prenatal exposure to PM_10_ we detected a reduced secretion of IL-10, especially after exposure during the last 3 days of pregnancy, and an increased secretion of IL-1β after exposure during the last three months of pregnancy. Maternal smoking during pregnancy was associated with reduced levels of IL-6 in cord blood. These findings indicate that exposure to regularly occurring in- and outdoor air pollution even in modest pollution areas can influence the expression of cytokines in cord blood serum, possibly modulating the maturing immune system very early in life.

### Comparison with literature

A number of epidemiological studies have addressed the effect of environmental tobacco smoke (ETS) and traffic-related air pollution with regard to onset, incidence, and severity of respiratory symptoms in children [2], [15], [16], [17], [18], [19], [20]. Less data exists on possible mechanisms and most studies have assessed acute effects of high levels of pollution on cytokine expression, mostly involving adults. In addition to the study by van Eeden *et al.* showing elevated blood cytokine concentrations in healthy adults after acute exposure to forest fires [6], an effect of high mean pollution levels on cytokine expression in healthy adults living in the Guadalajara metropolitan has also been shown [5].

Data of comparable effects in the growing child is very scarce. One German study found an effect of short-term indoor renovation activities on IL-8 and MCP-1 concentrations of six year old children [21]. Even less data exists regarding the developing immune system, with only few studies that have e.g. investigated the effect of indoor or outdoor air pollution on prenatal or early life immune development [12], [22], [23]. In line with our results one recent study found lower IL-6 and IL-10 responses to toll like receptor-2 ligation in cord blood of infants from smoking mothers [24]. Another study found decreased leukocyte subsets including myeloid precursor dendritic cells in cord blood of smoking mothers [13]. Also in parallel to our results, Hertz-Picciotto *et al.* have examined associations between air pollution and lymphocyte immunophenotypes in cord blood among 1397 deliveries in the Czech Republic. They detected decreases in CD3, CD4 and CD8 cells and increases in B-cells with increasing polycyclic aromatic hydrocarbon (PAH) and PM_2.5_ levels with an average of 24.8 µg/m^3^ after maternal exposure to air pollution in the last trimester of pregnancy [12]. A reduced expression of T-cells would explain the decrease of T-cell related cytokines such as observed for IL-10 and also IL-6 in our study. In contrast to these findings, Lehmann *et al.* detected increased Th2-cells in newborns after maternal exposure to volatile organic compounds (naphthalene or methylcyclopentane) and decreased Th1-cells after exposure to tetrachloroethylene [23]. Thus, it appears that specific exposures with different properties and molecular sizes influence T-cell responses in a specific manner.

In our study very specific and different time windows seemed to be relevant for changes of cord blood cytokines. This was also reported before by Herr *et al.*, who showed that both cord blood IgE levels and lymphocyte distribution were associated with exposure to air pollution during critical periods of pregnancy, highlighting the importance of specific time windows during prenatal immune development [25], [26].

In monkeys a change in blood levels of several cytokines was shown after exposure to ETS during pregnancy [27]. Interestingly, the authors were also able to show that the natural course of e.g. IL-10 in peripheral blood during the first year of life is altered in monkeys after and with continuing exposure to ETS compared to those without ETS exposure [27].

All this data not only reveals a possible mechanism for higher rates of respiratory symptoms in children of mothers who smoked during pregnancy [28] and who are exposed to high levels of air pollution [29], but especially suggests that prenatal exposure to environmental pollution impacts upon immune development with possible long-term consequences.

Several studies have shown an effect on TNF-α release by addition of PM_10_ particles to human monocytes *in vitro*, potentially providing a stimulus for production of pro-inflammatory mediators by lung epithelial cells [30], [31]. Van Eeden *et al.* described that circulated levels of IL-1β, IL-6 and GM-CSF were increased in macrophages exposed to EHC-93 (urban air dust from Ottawa particles, PM_10_) *in vitro*, and also in subjects exposed to high levels of PM_10_ *in vivo* [6], [32]. Similar to their and others' results [32], [33], we found increased levels of IL-1β in cord blood serum, which in concert with the decreased levels of IL-10 observed in our study highlights the hypothesis of a modulated and dysbalanced immune response upon prenatal exposure to PM_10_. In contrast to our findings, no influence on IL-10 was found in their study. This difference may be due to the measurement of cord blood serum in our study assessing peripheral blood systemic responses as compared to alveolar macrophages expressed in the lungs in the studies of van Eeden *et al.* [6], [14].

For several factors during pregnancy, such as maternal atopy, nutrition, exposure to environments with high microbial content and to aero-allergens, it has been shown that prenatal exposure can affect early life immune development including cytokine responses, lymphocyte proliferation and immunoglobulin levels [8], [9], [34], [35], [36], [37]. Furthermore, it is widely accepted that exposure to different stimuli during a specific perinatal window of susceptibility can subsequently contribute to several postnatal health problems, including decreased lung growth, increased rate of respiratory infections and childhood asthma [27], [38].

### Strengths, limitations and open questions

Our study has several strengths. Using un-stimulated cord blood in an area with modest pollution levels, the effect on a population level can be well estimated. With this model, we are able to extend known effects of ETS on immune modulation to PM_10_. Although performing multiple testing, similar trends for different time spans suggest a true association (Figure 1). On the other hand, as we were not able to examine T-regulatory cells or other T-cell subpopulations in this study, we cannot prove the specificity and function of cytokine secretion. Furthermore, cells in our study were not stimulated due to feasibility in a large birth cohort study. Thus, it might well be that, if assessed in supernatants of stimulated cells, immune responses could even point to a stronger pattern.

A limitation is the relatively high proportion of cytokine measurements below the detection limit. Thus, we had to dichotomize our outcomes (above vs. below the detection limit). However, such an outcome misclassification is rather expected to underestimate an association than to create a spurious association.

### Relevance

So far, it is well known that both ETS and air pollution is linked to the occurrence of allergy and asthma and further respiratory morbidity and mortality. Our study helps to elucidate immunological mechanisms behind data from epidemiological studies. Our analysis is the first one to address the effect of moderate levels of air pollution during the vulnerable phase of late pregnancy on cytokine concentrations in newborns on a population level, i.e. on a well-documented birth cohort of healthy term-born neonates [39]. This is of relevance to assess the putative link of exposure to both ETS and air pollution with allergy and asthma.

### Conclusion

In conclusion, our study shows that in- and outdoor exposure to modest levels of air pollution might impact upon neonatal immune responses. The further follow-up of this birth cohort will give us the opportunity to study how these early changes of the immune system, in combination with further exposures being assessed, relate to the occurrence of respiratory morbidity as well as lung growth and development. It is already of great importance to understand these early effects on the developing immune system. However it will be even more relevant to understand whether these changes show a sustained effect and whether it is thus warranted to identify individuals at risk for allergic diseases during an early stage.

## Materials and Methods

### Ethics statement

The Ethics Committee of the Region of Bern approved the study and written consent was obtained at enrolment.

### Study design

This prospective birth cohort study comprised 265 unselected, healthy neonates recruited prenatally between 1999 and 2005 in the region of Bern, Switzerland [39]. Exclusion criteria for the study were preterm delivery (<37 weeks), significant perinatal disease including respiratory distress and later diagnosis of chronic respiratory disease. Umbilical cord blood (n = 199) was obtained at the time of delivery. Potential risk factors (sociodemographic status, smoke exposure and parental atopic disease) were assessed by interviews using standardized questionnaires.

### Exposure assessment

We assessed maternal exposure to outdoor and indoor air pollution over different time periods during the last trimester of pregnancy. Air pollution data included daily mean levels of PM_10_ for the period from January 1999 to December 2005. Air pollution was measured at the monitoring station of Payerne (part of the Swiss National Air Pollution Monitoring Network NABEL), which lies within the study area and reflects temporal variability of air pollutants during the study period. We used these regional data to calculate the mean exposure to PM_10_ for each subject and the following time periods during pregnancy: for the last trimester in total, as this has been shown before to influence lung development in the fetus [40] and for the last 21, 14, 7, 3 and 1 days of pregnancy, as those time periods seem to be sensible for a possible influence of air pollution on immune development [12].

As a proxy for traffic-related air pollution exposure, we computed distance from the family's home coordinates to the closest major road of at least 6 meter (1^st^ class road) in width. This was done analogous to another Swiss cohort study (for street categories of the VECTOR25 see Appendix Table of [41]. Calculations were performed with a geographic information system (GIS, ArcGIS, version 9, Environmental Systems Research Institute, Redlands, USA). Addresses were geo-coded using the building registry of the Swiss Federal Statistical Office, and street information was obtained from the VECTOR25 map of the Swiss Federal Office of Topography (www.swisstopo.ch).

As a proxy for exposure to indoor air pollution, we used questionnaire data on smoking habits, cooking facilities (gas oven) and open fireplaces in the homes of the parents. Data on antenatal smoking habits of the mothers were validated using cotinine levels in the first urine of the newborn (gas-liquid chromatography; ITS, Lausanne, Switzerland).

### Measurement of cord blood cytokines

Cord blood serum was collected after delivery from the umbilical vein from trained midwifes and samples were processed as previously described [34]. Serum was centrifuged and frozen until analysis. Serum was aliquoted in duplicate into 96-well plates (50 µl/well), which were precoated with cytokine specific antibody. The human Cytokine Multiplex Assay Kit (including IL-1ß, IL-6, IL-10, GM-CSF, MCP-1, TNF-α) was used according to the manufacturer's instructions (Bio-Rad, Munich, Germany). The detection limits (pg/ml) of the assay were 0.6 for IL-1ß, 0.4 for IL-6, 0.4 for IL-10, 0.2 for GM-CSF, 0.5 for MCP-1 and 1.4 for TNF-α.

### Statistical analysis

For descriptive purpose summary statistics of blood cytokine levels were calculated using the robust regression on order statistics (ROS) method, which allows to include non-detects [42].

We conducted logistic regression analyses and dichotomized the outcomes (cord blood cytokine concentrations) into detectable vs. non-detectable according to the detection limit of the respective cytokine levels. For all regression models the associations were calculated (a) unadjusted, (b) adjusted for known confounders (maternal smoking in pregnancy, gender, gestational weight) and (c) additionally adjusted for possible confounders (social class, gestational age, maternal atopy). Data analyses were performed using STATA version 10 for Windows (STATA Corporation, College Station, USA).
